# Preparation and Characterization of Graphene Oxide/Carbon Nanotube/Polyaniline Composite and Conductive and Anticorrosive Properties of Its Waterborne Epoxy Composite Coatings

**DOI:** 10.3390/polym16182641

**Published:** 2024-09-19

**Authors:** Yufeng Li, Shibo Liu, Feng Feng, Yiming Li, Yahui Han, Xinyang Tong, Xiaohui Gao

**Affiliations:** 1 College of Chemistry and Chemical Engineering, Qiqihar University, Qiqihar 161006, Chinagxh1172@163.com (X.G.); 2 College of Light Industry and Textile, Qiqihar University, Qiqihar 161006, China

**Keywords:** graphene oxide (GO), carbon nanotube (CNT), polyaniline (PANI), waterborne epoxy (WEP), electrical conductive, anticorrosive properties

## Abstract

The organic coating on the surface is common and the most effective method to prevent metal materials from corrosion. However, the corrosive medium can penetrate the metal surface via micropores, and electrons cannot transfer in the pure resin coatings. In this paper, a new type of anticorrosive and electrically conductive composite coating filled with graphene oxide/carbon nanotube/polyaniline (GO/CNT/PANI) nanocomposites was successfully prepared by in situ polymerization of aniline (AN) on the surface of GO and CNT and using waterborne epoxy resin (WEP) as film-forming material. The structure and morphology of the composite were characterized using a series of characterization methods. The composite coatings were comparatively examined through resistivity, potentiodynamic polarization curves, electrochemical impedance spectroscopy (EIS), and salt spray tests. The results show that the GO/CNT/PANI/WEP composite coating exhibits excellent corrosion resistance for metal substrates and good conductivity when the mass fraction of GO/CNT/PANI is 3.5%. It exhibits a lower corrosion current density of 4.53 × 10^−8^ A·cm^−2^ and a higher electrochemical impedance of 3.84 × 10^6^ Ω·cm^2^, while only slight corrosion occurred after 480 h in the salt spray test. The resistivity of composite coating is as low as 2.3 × 10^4^ Ω·cm. The composite coating possesses anticorrosive and electrically conductive properties based on the synergistic effect of nanofillers and expands the application scope in grounding grids and oil storage tank protection fields.

## 1. Introduction

Metals and alloys have been widely used in various fields with the advancement of science and technology [1]. However, they are susceptible to chemical or electrochemical corrosion in complex working environments, which reduces the service life and causes economic losses and accidents [2]. One of the most widely and effective ways to solve metal corrosion problems is to apply an organic coating on the metal surface, along with some anticorrosive fillers. Among others, waterborne epoxy resin has been widely acknowledged and has become more and more important in anticorrosion coatings for its excellent mechanical properties and stability, especially its non-toxic and low VOC advantages [3,4]. In addition, suitable anticorrosion functional fillers could further improve the anticorrosive properties of its composite coatings.

The coating must have excellent electrically conducting properties besides good corrosion resistance in certain fields, such as electromagnetic/radio frequency interference (EMI/RFI) shielding, electrostatic and lightning strike protection, especially grounding grids and oil storage tank protection. Therefore, it is important to improve the conductive and anticorrosion properties of the coating simultaneously by adding functional fillers with conductive and anticorrosion properties to the composite coating. This approach expands the potential application range of polymer coatings. Up to now, there are three kinds of fillers used in conductive and anticorrosion composite coatings. Firstly, metal fillers, such as gold [5], silver [6], copper [7], zinc [8], and zinc oxide [9] as well, have been widely studied for electrically conducting properties, but their application is limited by the high cost or low chemical stability. Secondly, carbon fillers, such as carbon black [10] and carbon fiber [11], have the advantages of high chemical stability, a wide range of uses, and low prices. However, traditional carbon conductive coatings have limitations in terms of conductive performance. In recent years, novel carbon system fillers like graphene [12] and carbon nanotubes (CNT) [13] have attracted more and more attention due to their unique two-dimensional nanosheet structure and spatial conductive network, which further improve the anticorrosion and conductive properties of composite coatings. However, the compatibility between carbon materials and polymer coatings remains a challenge. Thirdly, conductive polymers such as polyaniline (PANI) [14] and polypyrrole [15] offer adjustable conductivity and passivation of metal surfaces and also have high compatibility with polymer resins. However, they are less conductive than metals and carbon materials.

It is a good idea to combine graphene oxide (GO), CNT, and PANI because there are many synergy effects among them, and they are beneficial to improving the conductivity and corrosion resistance of the coating. More specifically, GO and CNT are derivatives of graphene [16]. CNT walls resemble rolled-up graphite-like sheets with strong covalent sp^2^ bonds. Adding a small amount of CNT to the coating can improve the mechanical strength, wear resistance, corrosion resistance, and electrical conductivity. GO possesses a large number of hydrophilic functional groups such as carboxyl, carbonyl, hydroxyl, and epoxy groups on the surface compared to graphene [17], which allows better dispersion in water and various solutions. The distinctive two-dimensional structure of GO enables it to generate a “labyrinth effect” within the coating. This effect efficiently restrains the spread of corrosive substances in the coating [18]. The high aspect ratio of CNT and the strong van der Waals force between GO layers cause CNT and GO to agglomerate, which negatively affects the distribution of fillers within the coating and coating performance. To improve the dispersion of fillers in the coating, the filler could be modified or compounded with other fillers. Xu et al. [19] demonstrate that CNT is distributed among graphene sheets and forms a three-dimensional structure that can prevent the accumulation of graphene sheets. In a study by Chen et al. [20], CNT and reduced graphene oxide (rGO) were added to polydimethylsiloxane to utilize the barrier effect of graphene, which effectively slows down the agglomeration of CNT. The synergy between rGO and CNT addresses the poor dispersion of nanocarbon materials. Additionally, the combination of rGO and CNT forms a three-dimensional conductive network within the polymer, significantly enhancing the polymer coating’s conductivity. As the most studied conductive polymer, PANI has been widely used as a conductive component in conductive and anticorrosive coatings due to its ease of synthesis, unique redox properties, adjustable conductivity, and chemical stability [21]. Terminal amino groups of PANI could react with the epoxy matrix during curing reactions, which guarantees interfacial adhesion between carbon materials and epoxy resin [22]. However, there are few reports on preparing composite coatings based on the synergistic effect of GO, CNT, and PANI.

In this paper, the graphene oxide/carbon nanotube/polyaniline (GO/CNT/PANI) composite was prepared by in situ polymerization with GO, acid-treated CNT (Ac-CNT) and aniline (AN) as raw materials, and following the new type GO/CNT/PANI/WEP composite coating with electrically conductive and anticorrosive properties was prepared with waterborne epoxy resin (WEP) as film-forming material. This study aims to improve anticorrosive and electrically conductive properties simultaneously by creating a three-dimensional conductive network in GO/CNT/PANI composite. In this process, the transmission speed of electrons and dispersion of nanofiller were enhanced by investigating the optimal ratio of GO and CNT. Meanwhile, the conductivity of GO, the corrosion resistance of CNT, and the adhesion between carbon materials and epoxy resin were improved by coating PANI over the surface of GO and CNT. Moreover, the addition of GO/CNT/PANI into WEP can effectively fill the pores formed during the curing process, and this “labyrinth effect” efficiently restrains the spread of corrosive substances in the coating. The nanocomposite filler could significantly enhance the conductivity, compactness, and corrosion resistance of GO/CNT/PANI/WEP composite coating by the synergistic effect of GO, CNT, and PANI.

## 2. Materials and Methods

### 2.1. Materials

Carbon nanotubes (CNT) were procured from Carbonene Technology Co., Ltd. (Shenzhen, China). Natural graphite was obtained from Shuitian Materials Technology Co., Ltd. (Shanghai, China). Aniline (AN) and potassium permanganate (KMnO_4_) were supplied from the Reagent Chemical Plant (Harbin, China). Ammonium persulfate (APS), sulfuric acid (H_2_SO_4_, 98%), nitric acid (HNO_3_, 68%), hydrochloric acid (HCl, 36%), and sodium chloride (NaCl) were provided by Kaitong Chemical Reagent Co., Ltd. (Tianjin, China). Ethanol (C_2_H_5_OH, 99.5%) and hydrogen peroxide were provided by Kermel Chemical Reagent Co., Ltd. (Tianjin, China). Waterborne epoxy resin (WEP) and curing agent (AB-HGC) were supplied from Anbang New Material Development Co., Ltd. (Zhejiang, China). The deionized water was used throughout the experiments.

### 2.2. Preparation of Acidified Carbon Nanotubes (Ac-CNT)

A total of 0.4 g CNT was added to a three-necked round-bottomed flask and dispersed by ultrasound for 60 min. Next, sulfuric acid (98%, 30 mL) and nitric acid (68%, 10 mL) were added and dispersed by ultrasound for another 30 min. Then, the flask was equipped with a reflux condenser and thermometer and placed in a magnetic stirrer with a thermostat. The temperature was maintained at 60 °C for 6 h, after which the slurry was cooled to room temperature (at ~25 °C) and poured out, filtered, and washed with distilled water several times until a neutral pH was attained. Finally, it was freeze-dried in a vacuum freeze dryer for 48 h.

### 2.3. Preparation of Graphene Oxide (GO)

The graphene oxide was prepared based on an improved Hummer’s method [23].

### 2.4. Preparation of CNT/PANI Nanocomposites

A total of 0.1 g Ac-CNT and 20 mL HCl (1 mol/L) were added to a three-necked round-bottomed glass flask and dispersed by ultrasound for 1 h. The resulting Ac-CNT dispersion was then mixed with 0.02 g AN monomer, and the temperature was maintained at 0–5 °C for 4 h. A total of 0.49 g APS was dissolved in 5 mL HCl solution (0.1 mol/L) and then slowly added into the above mixture drop by drop under constant magnetic stirring at 0–5 °C. The temperature was maintained at 0–5 °C for 24 h, then the black solid product was washed with deionized water several times until the pH reached 7. Finally, it was freeze-dried in a vacuum freeze dryer for 48 h.

### 2.5. Preparation of GO/CNT/PANI Nanocomposites

**Figure 1:**
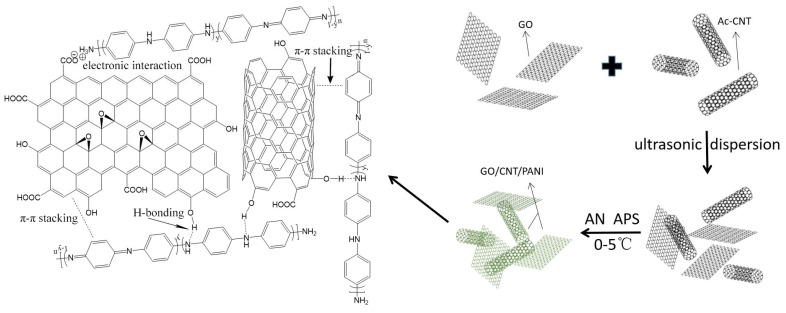
Schematic illustration of synthesis of GO/CNT/PANI composites.

The prepared route of GO/CNT/PANI nanocomposites is shown in Figure 1. A total of 0.1 g Ac-CNT and HCl (1 mol/L, 20 mL) was added to a three-necked round-bottomed glass flask and dispersed by ultrasound for 1 h. Then 0.02 g GO was added and dispersed by ultrasound for another 60 min. A total of 0.02 g AN was added and maintained at 0–5 °C for 4 h, and the APS solution (0.49 g APS dissolved in 5 mL 0.1 M HCl) was then slowly added dropwise. After this, the temperature was maintained at 0–5 °C for 24 h, and the slurry was poured out, filtered, and washed with deionized water several times until the pH reached 7. After pre-freezing, it was freeze-dried in a vacuum freeze dryer for 48 h.

### 2.6. Coating Preparation

The Q235 steel was polished with 500, 1000, and 2000 grit sandpaper to clean the surface and remove possible surface impurities. A total of 4 g WEP and 0.6 g water were added to a disposable plastic cup and stirred uniformly. Then, an appropriate amount of filler was weighed and dispersed in WEP emulsion, and the mixture was stirred at speed for 30 min to achieve a uniform dispersion of filler paint. Then, 0.625 g of curing agents were added to the mixture. Finally, the mixture was evenly coated on the surface of the clean Q235 steel sheet, placed at room temperature for 4 h, and dried in 40 °C ovens for 48 h. Additionally, the average thickness of the dried coating was kept at 200 ± 10 μm.

To compare and discuss, a series of composite coatings were prepared by using the same method mentioned above, and the formulations of all the samples were presented in Table 1 and Table 2. Table 1 lists the formulations of CNT/WEP composite coatings with different CNT contents from 0.5 to 6.0% and labeled by A1~A11. Table 2 lists the formulations of CNT/PANI/WEP composite coatings with different AN/CNT from 5.0 to 20.0% and is labeled by B1~B4, and the formulations of GO/CNT/PANI/WEP composite coatings with different GO/(AN + CNT) from 5.0 to 25.0% are labeled by C1~C5.

### 2.7. Characterization

The functional groups were analyzed by Fourier transform infrared (FT-IR) spectroscopy (Perkin Eimer, Spectrum one, Waltham, MA, USA), with a scanning range of 4000~500 cm^−1^. The crystal structures of prepared materials were characterized by X-ray diffraction (XRD) with an X-ray diffractometer (BRUKER-AXS, D8, Karlsruhe, Germany). The surface morphology of the prepared materials was characterized by scanning electron microscope (SEM) (HITACHI, S-4300, Shinagawa-ku, Japan) and transmission electron microscope (TEM) (HITACHI, H-7650, Shinagawa-ku, Japan). The electrochemical impedance spectroscopy (EIS) and potentiodynamic polarization measurements were adopted to detect the corrosion protection performance of each coating in a 3.5% NaCl solution at room temperature. EIS tests were carried out at an electrochemical workstation (Gamry, Interface1000, Warminster, PA, USA) with a three-electrode system including the working electrode (Q235 steel), the counter electrode (platinum), and the reference electrode (Ag/AgCl). The frequency was in the range of 10^5^–10^−2^ Hz with an amplitude of ±5 mV. The potentiodynamic polarization curves were obtained with a sweep rate of 0.5 mV/s. The corrosion resistance of the coatings was also tested with the salt-spray test chamber (OL-T-60, Shenzhen, China). The conductivity was tested with a four-probe meter (RTS-5, RTS-8, Guangzhou, China). Flexibility, adhesion, pencil hardness, and impact resistance of coatings were evaluated according to the China standards GB/T 6742-2007 [24], GB/T 9286-2021 [25], GB/T 6739-2006 [26], and GB/T 1732-1993 [27], respectively.

## 3. Results and Discussions

### 3.1. Microstructure Analysis

#### 3.1.1. FT-IR Analysis

**Figure 2:**
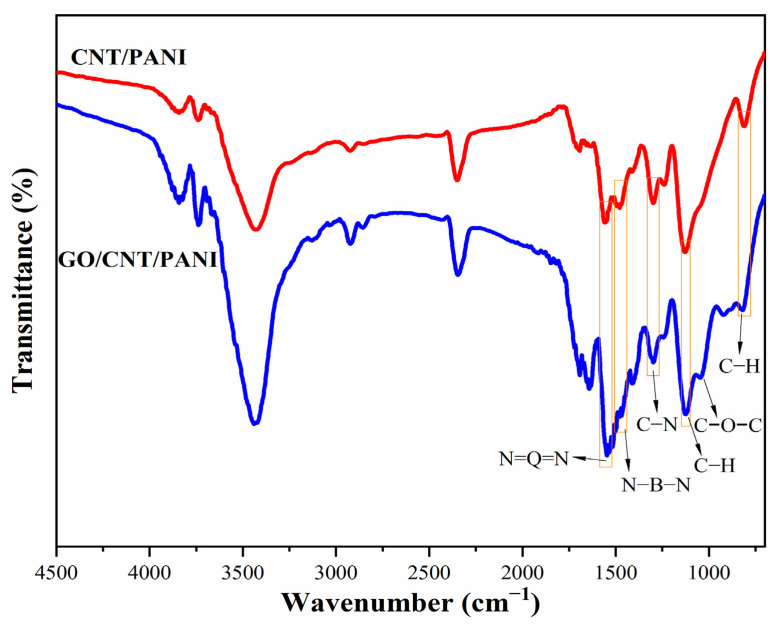
FT-IR spectra of CNT/PANI and GO/CNT/PANI composites.

Figure 2 and Figure S1 show the FT-IR spectra of PANI, Ac-CNT, CNT/PANI, and GO/CNT/PANI. The adsorption peaks of O-H [28], C=O, and C-O [29] indicate the presence of carboxyl and hydroxyl groups in Ac-CNT. The adsorption peaks at 1046 cm^−1^ can be observed in the FT-IR spectra of GO/CNT/PANI, indicating the introduction of C-O-C in CNT/PANI by adding GO. For CNT/PANI and GO/CNT/PANI, the peak exhibited at 1561 cm^−1^ and 1474 cm^−1^ attributed to the presence of quinoid and benzenoid rings in the emeraldine salt [30]. The absorption peak at 1287 cm^−1^ is caused by the C-N stretching vibration [31], and the absorption peak at 1106 cm^−1^ and 786 cm^−1^ corresponds to the stretching vibration peak and bending vibration of C-H, respectively [32]. The characteristic peaks of PANI can be observed in the FT-IR spectra of CNT/PANI and GO/CNT/PANI, confirming that aniline is converted into PANI in carbon materials by in situ polymerization.

**Figure 3:**
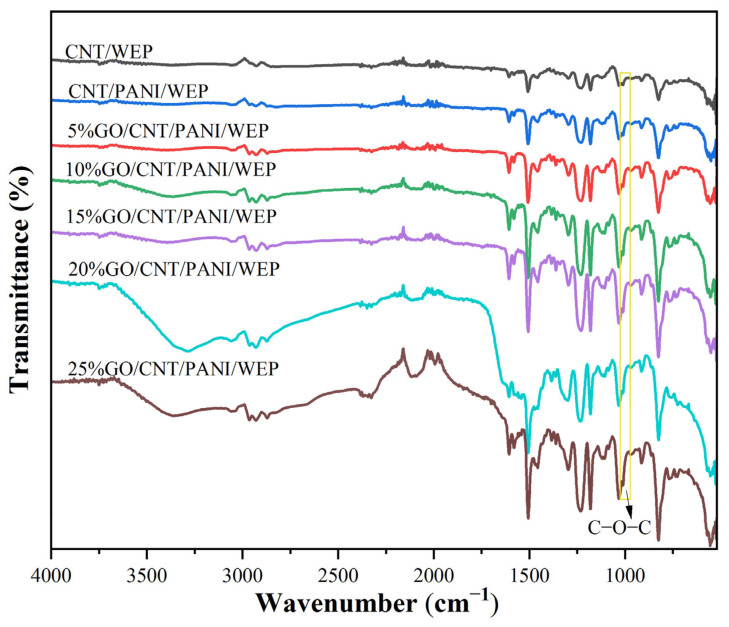
FT-IR spectra of different composite coatings.

Figure 3 shows the FT-IR spectra of CNT/WEP composite coating, CNT/PANI/WEP composite coating, and GO/CNT/PANI/WEP composite coatings with different content of GO. The intensity of the adsorption peaks of C-O-C at about 1011 cm^−1^ increased as the content of GO increased, demonstrating that the change in content of GO could reflect in composite coatings and consequently affect the performance of composite coatings differently.

#### 3.1.2. XRD Analysis

**Figure 4:**
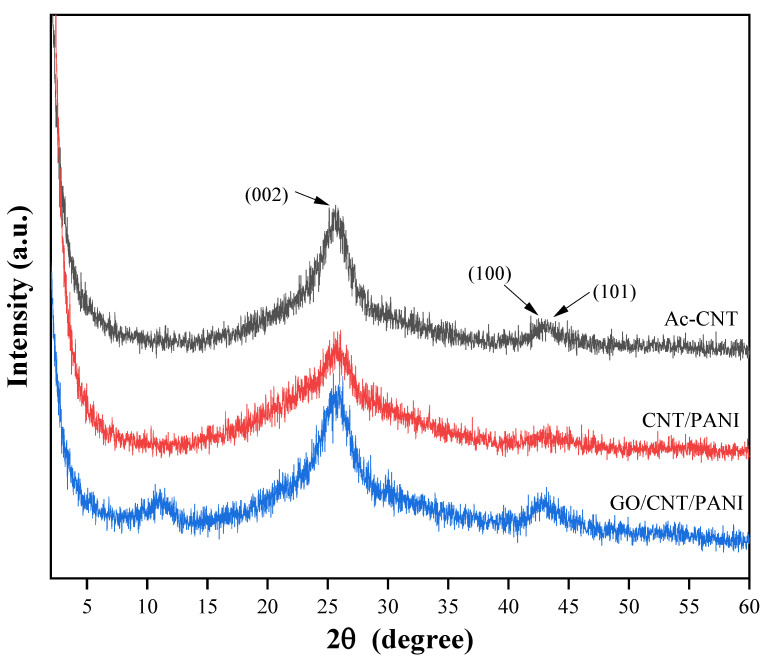
XRD patterns of Ac-CNT, CNT/PANI, and GO/CNT/PANI nanocomposites.

Figure 4 and Figure S2 exhibit XRD patterns of GO, PANI, Ac-CNT, CNT/PANI, and GO/CNT/PANI. From the figure, it can be seen that the XRD curve of GO has a sharp broad diffraction peak at 2θ = 11.3°, which belongs to the diffraction peak of the crystalline surface of GO (002), and the small diffraction peak at 2θ = 42.3° is the diffraction peak of the specular surface of GO (101), which indicates that the oxidation process leads to extension in the interlayer separation [33,34]. In the XRD curve of PANI, 2θ of 9.1°, 14.8°, 20.4°, 25.4°, 26.8°, and 29.2° represent the crystal diffraction peaks of PANI (001), (011), (020), (200), (121), and (002) crystal diffraction peaks, respectively [35]. In the XRD curve of Ac-CNT, the broad and robust diffraction peaks at 2θ = 25.6° belong to the characteristic diffraction peaks of the crystalline surface of Ac-CNT (002), and the broad and small diffraction peaks at 2θ = 42.7° and 43.2° belong to the characteristic diffraction peaks of the crystalline surface of Ac-CNT (100) and (101) [36]. In the XRD curves of CNT/PANI and GO/CNT/PANI, the characteristic diffraction peaks of PANI appeared. Meanwhile, the characteristic peaks on the (100) and (101) crystal surfaces of Ac-CNT in the XRD curves of CNT/PANI and the characteristic peaks on the (002) crystal surface of GO in the XRD curves of GO/CNT/PANI were weakened after introducing PANI, which indicates that there is an interaction of PANI through the amines with the carboxyl groups on the surfaces of GO and Ac-CNT. These changes also prove that the PANI is tightly encapsulated on the surfaces of GO and Ac-CNT [37].

### 3.2. Morphology Analysis

**Figure 5:**
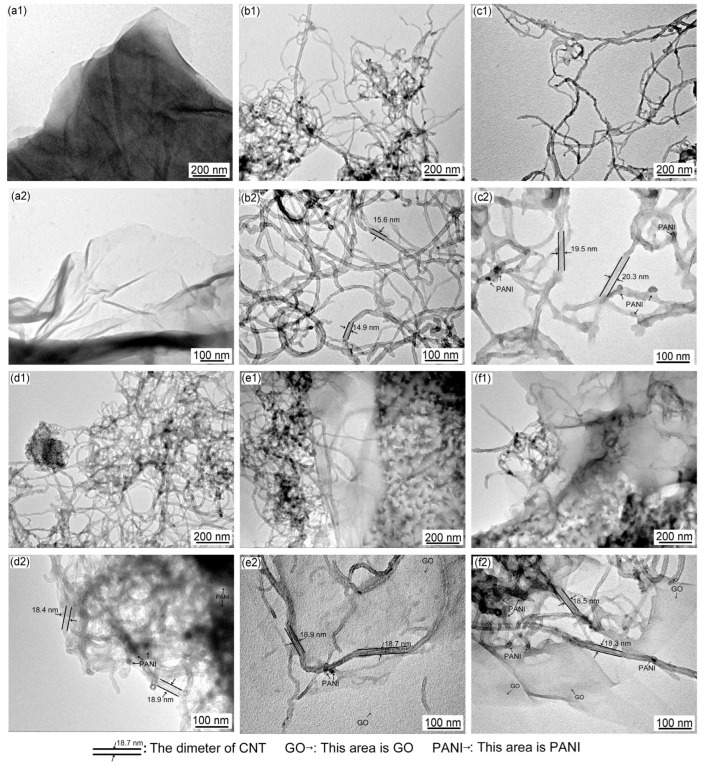
TEM images of (**a1**,**a2**) GO, (**b1**,**b2**) Ac-CNT, (**c1**,**c2**,**d1**,**d2**) CNT/PANI, and (**e1**,**e2**,**f1**,**f2**) GO/CNT/PANI.

TEM images of GO, Ac-CNT, CNT/PANI, and GO/CNT/PANI are shown in Figure 5. Figure 5(a1,a2,b1,b2) show that the surface of GO and the wall of Ac-CNT are smooth and nearly flat. Figure 5(c1,c2) shows that the roughness of the Ac-CNT wall increases when PANI is combined with CNT to form CNT/PANI composite fibers, which proves that PANI was coated on the surface of Ac-CNT through in situ oxidative polymerization. The PANI prevents the agglomeration of Ac-CNT because PANI is coated on the surface of Ac-CNT. However, CNT/PANI will agglomerate when PANI is added too much, as shown in Figure 5(d1,d2). Therefore, incorporating an appropriate amount of PANI could improve the performance of the composite filler.

Figure 5(e1,e2) show that PANI was coated on the surface of GO and Ac-CNT through in situ oxidative polymerization to form GO/CNT/PANI composites. It can be observed that the surface of GO is also uniformly coated by PANI particles, which is beneficial to prevent the agglomeration of PANI. Meanwhile, the presence of CNT and PANI prevents the agglomeration of GO. However, with the same result as above, the distribution of PANI on the GO surface is uneven when GO is added too much, as shown in Figure 5(f1,f2), which will aggravate the agglomeration of composite fillers [38].

As shown in Figure 5(b2), the diameter of Ac-CNT is about 15 nm. The diameter of Ac-CNT increased to about 20 nm after composite with PANI (Figure 5(c2)), confirming that PANI was coated on the surface of Ac-CNT. Similarly, the diameter of Ac-CNT increased to about 19 nm after composite with PANI and GO, and some dark spots formatted around (Figure 5(e2)) confirm that PANI was coated on the surface of Ac-CNT and GO. Nevertheless, agglomeration occurred, and the diameter of Ac-CNT decreased when GO was added too much (Figure 5(d2,f2)).

**Figure 6:**
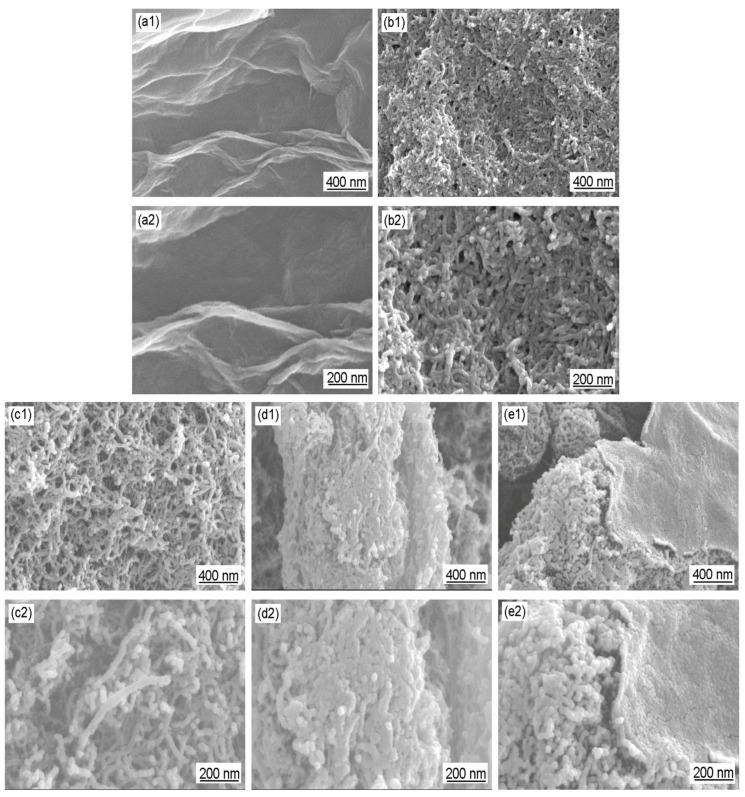
SEM images of (**a1**,**a2**) GO, (**b1**,**b2**) Ac-CNT, (**c1**,**c2**,**d1**,**d2**) CNT/PANI, and (**e1**,**e2**) GO/CNT/PANI.

Figure 6 shows the SEM images of GO, Ac-CNT, CNT/PANI, and GO/CNT/PANI. Figure 6(a1,a2,b1,b2) show that the surface of GO and the wall of Ac-CNT are smooth. By comparing Figure 6(c1,c2,d1,d2), it can be seen that the SEM results are consistent with the TEM results, i.e., PANI was coated on the surface of Ac-CNT uniformly, but a large amount of PANI leads to the agglomeration of CNT/PANI. Figure 6(e1,e2) shows that the surfaces of Ac-CNT and GO are uniformly coated by PANI particles. Moreover, one-dimensional CNTs are inserted into two-dimensional graphene sheets and constitute three-dimensional composite materials. This structure could further improve the conductivity of GO, the corrosion resistance of CNT, and the compatibility between carbon materials and polymer coatings as well.

In conclusion, SEM and TEM demonstrated that the appropriate amount of PANI can be uniformly coated on the surfaces of CNT and GO, and there exists an optimal ratio of the amount of CNT and GO. The synergistic effect among CNT, GO, and PANI can be fully realized if these conditions are satisfied.

### 3.3. Dispersion Stability

#### 3.3.1. The Aggregation and Sedimentation Process

**Figure 7:**
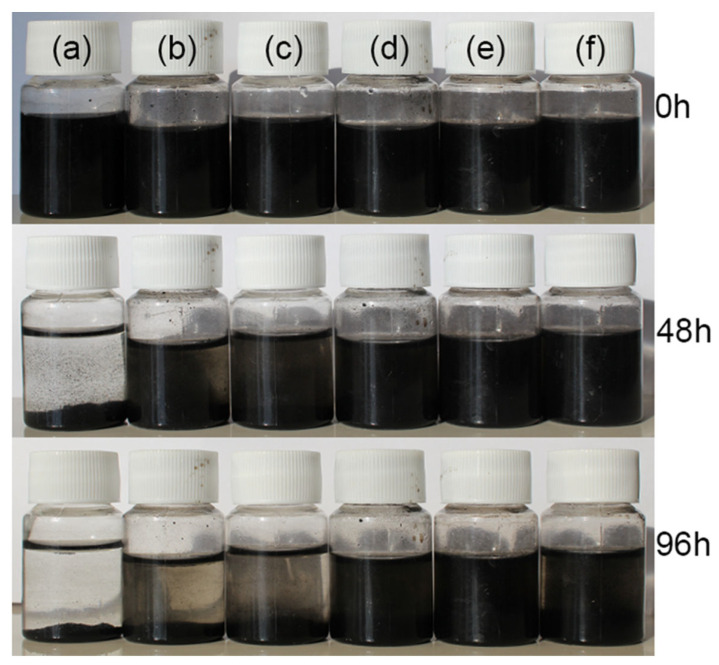
The dispersion stability photos of (**a**) CNT/PANI and GO/CNT/PANI composite fillers with different GO contents (**b**) 5%, (**c**) 10%, (**d**) 15%, (**e**) 20%, and (**f**) 25%.

Figure 7 displays the aggregation and sedimentation process photos for the CNT/PANI composite (a) and GO/CNT/PANI composite filler in water and compares the dispersion stability of GO/CNT/PANI composites in water with varying GO concentrations (b) 5%, (c) 10%, (d) 15%, (e) 20%, and (f) 25%. The experiments indicated that the dispersion of composites in water improved gradually with an increase in GO content as GO/CNT/PANI composites were prepared. The best dispersion of the filler in water was achieved when the GO mass was 20% of CNT and AN, but the dispersion of the composites decreased when too much GO was added.

The results demonstrated that adding an appropriate amount of GO can minimize the agglomeration of nanofillers in water. The following factors may cause this phenomenon: GO comprises numerous hydrophilic groups, including carboxyl and hydroxyl groups, and GO has a barrier effect on CNT agglomeration. Adding the appropriate quantity of GO facilitates the dispersal of the composite filler in water [39]. However, GO is prone to agglomeration due to Van der Waals forces; if an excessive amount of GO is added, it can lead to a decline in the dispersion of the composite material in water [40].

#### 3.3.2. Morphology of Composite Coating

**Figure 8:**
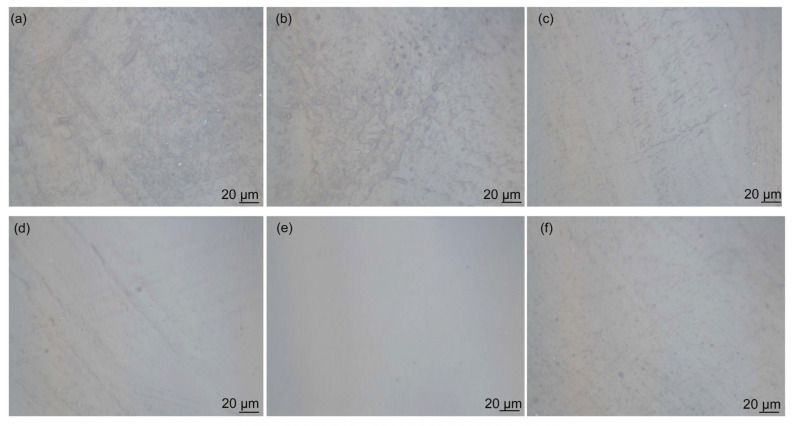
Optical microscopy images of surface morphology of (**a**) CNT/WEP, (**b**) CNT/PANI/WEP and GO/CNT/PANI/WEP coatings with different GO contents ((**c**) 5%, (**d**) 15%, (**e**) 20%, and (**f**) 25%).

Figure 8 shows the effect of GO on the dispersion of GO/CNT/PANI composite filler in WEP composite coatings. For comparison, Figure 8a,b shows the morphology of CNT/WEP and CNT/PANI/WEP composite coatings. The amounts of GO in the filler shown in Figure 8c–f correspond to 5%, 15%, 20%, and 25% of CNT and AN, respectively. It can be observed that there are numerous filamentary agglomerations of CNT in the coating without or at low levels of GO content (Figure 8a–c). The agglomeration phenomenon decreases as the GO content increases (Figure 8d), and the GO/CNT/PANI composite filler exhibited the most uniform distribution within the coating at a GO content of 20% (Figure 8e). However, continuing to increase the amount of GO in the filler results in the accumulation of GO flakes, which in turn leads to inadequate dispersion of the GO/CNT/PANI composite filler in the coating, and spotted aggregation appeared in the composite coating, as shown in Figure 8f. The results indicate that the distribution of filler in water aligns with the distribution of filler in the aqueous coating. The nanofiller in the WEP composite coating is more uniform when the mass fraction of GO is 20% of CNT and AN.

**Figure 9:**
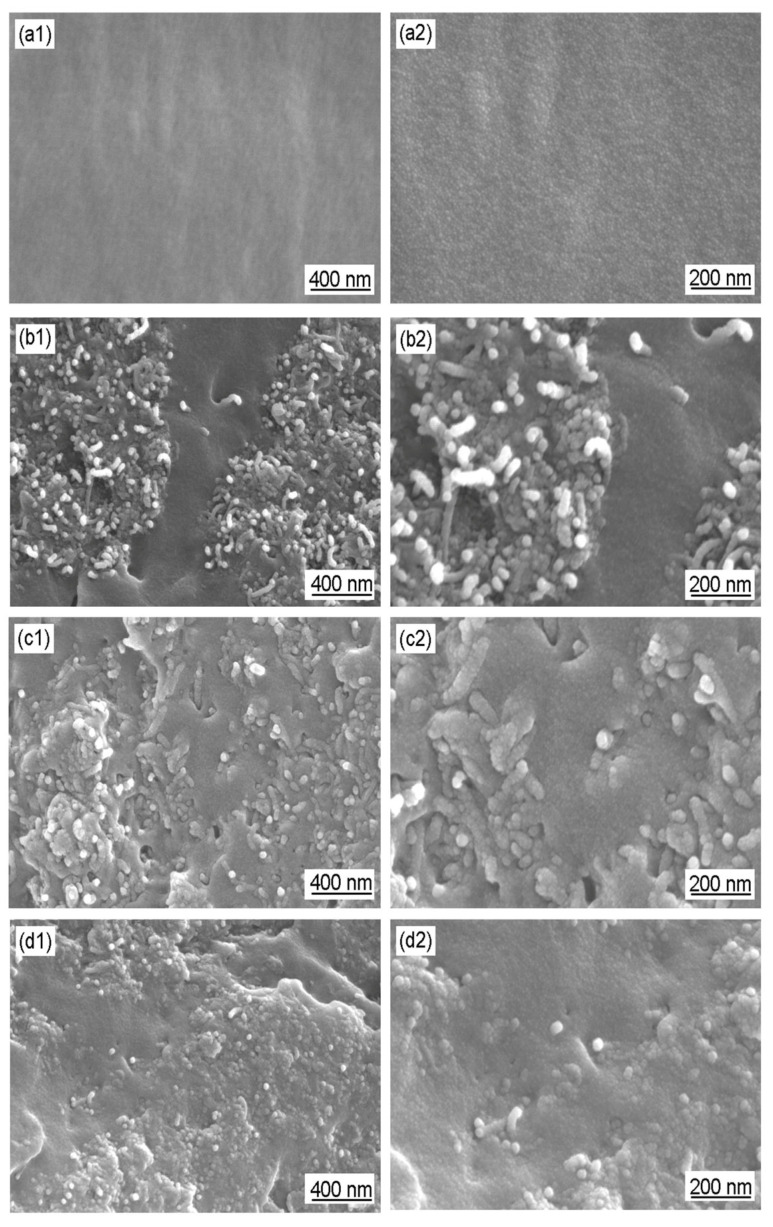
SEM images of a cross-section of (**a1**,**a2**) WEP coating and (**b1**,**b2**) CNT/WEP, (**c1**,**c2**) CNT/PANI/WEP, and (**d1**,**d2**) GO/CNT/PANI/WEP composite coatings.

For deeper analysis of the coatings, SEM images of a cross-section of WEP coating and CNT/WEP, CNT/PANI/WEP, and GO/CNT/PANI/WEP composite coatings are shown in Figure 9. As a polymer matrix, WEP coating exhibits homogeneous morphology (Figure 9(a1,a2)). CNT is distributed in the polymer matrix with an agglomerate state after blending with WEP (Figure 9(b1,b2)). For CNT/PANI/WEP composite coating, the agglomeration of CNT is prevented by PANI to a certain extent (Figure 9(c1,c2)). For GO/CNT/PANI/WEP composite coating, GO/CNT/PANI composite exhibits a more uniform distribution within the polymer matrix due to the introduction of GO (Figure 9(d1,d2)). The GO/CNT/PANI composite has great dispersibility in coatings and can improve the mechanical performances, electrical conductivity, and anticorrosive properties of composite coatings.

### 3.4. Contact Angle

**Figure 10:**
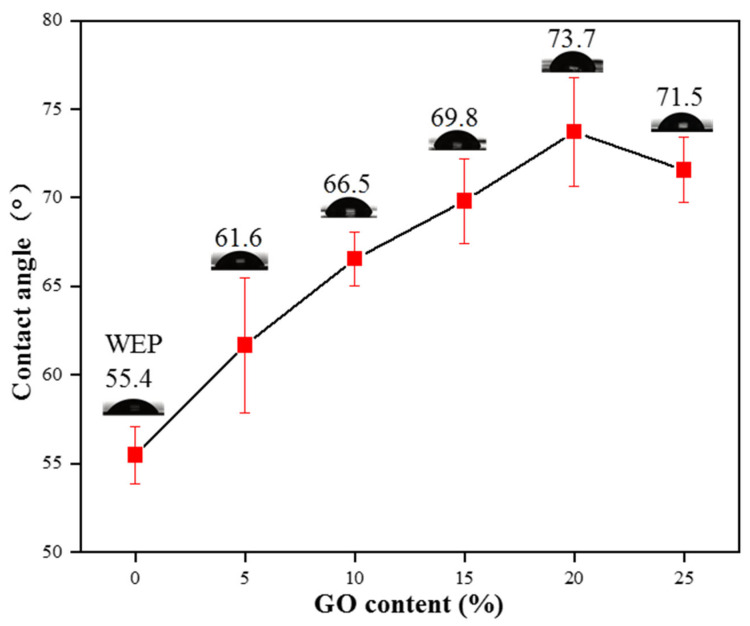
Hydrophobicity of WEP and GO/CNT/PANI/WEP composite coatings with different GO contents.

Hydrophobicity can also enhance corrosion resistance due to decreased water/surface interactions [41]. Figure 10 displays the level of hydrophobicity present in WEP and GO/CNT/PANI/WEP at different GO concentrations. From the graph, it is evident that the coating’s hydrophobicity increases gradually with the percentage of GO in the GO/CNT/PANI composite filler, and the contact angle of coatings peaks at 73.7° when the filler contains 20% GO. Combining the findings from Figure 7 and Figure 8, the findings illustrate that the filler is optimally dispersed within the coating, which increases its ability to hydrophobicity and is the primary reason for the maximum contact angle of the coating. Continuing to escalate the amount of GO can lead to poor dispersion of the composite filler in the coating, resulting in a decrease in the hydrophobicity of the coating, which negatively affects the anticorrosive performance of the coating.

### 3.5. Resistivity Measurement

**Figure 11:**
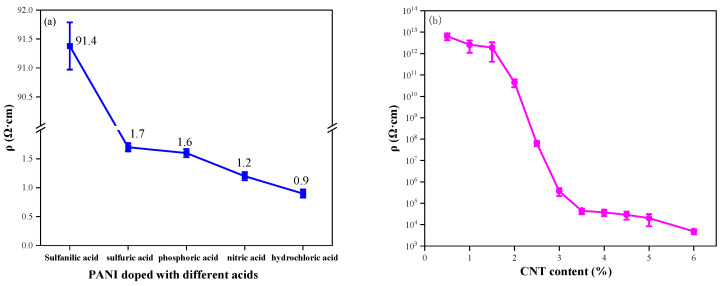

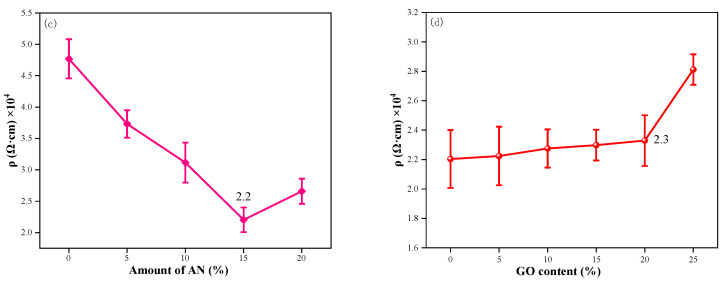
The electrical conductivity of (**a**) PANI doped with different acids; (**b**) CNT/WEP composite coatings with different content of CNT; (**c**) CNT/PANI/WEP composite coatings with different components of AN; (**d**) GO/CNT/PANI/WEP composite coatings with different components of GO.

Figure 11a compares the resistivity of PANI samples prepared with various doping acids. PANI prepared with inorganic acid doping exhibits lower resistivity than that prepared with organic acid doping. Among inorganic acid-doped samples, PANI prepared with hydrochloric acid doping displays the lowest resistivity at only 0.9 Ω·cm. Therefore, choosing hydrochloric acid-doped PANI for nanocomposite preparation with carbon materials is more favorable for enhancing the conductivity of composite coatings.

Figure 11b shows the resistivity data for CNT/WEP composite coatings loaded with different amounts of CNT. It is proved that the conductive path in the coating forms a conductive network when the additional amount of CNT is 3.5%. It is clear from Figure 11b that the introduction of small amounts of CNT into the WEP coating does not result in a significant change in the resistivity of the composite coating. The resistivity of the composite coatings decreased significantly as the CNT content increased from 1.5% to 3.5%, and subsequently, the resistivity of the composite coatings did not change much as the CNT content in WEP continued to increase. The resistivity of a composite coating is closely related to the conductive network within the coating, which is influenced by the electrical conductivity of the fillers and the direct contact conductance at the microscale [42]. For low CNT levels in composite coatings, the network path cannot be formed due to the sparse quantity of CNT, resulting in the composite coating remaining in an electrically insulating state. Continuing to increase the amount of CNT in the composite coating, CNT can form electrical paths in the composite coating, leading to a rapid decrease in the resistivity of the coating as the number of conductive pathways in the coating increases. The resistivity changes tend to stabilize when the electrical paths in the coating form a conductive network after the CNT content increases to a certain amount. The minimum filler volume fraction at which this phase transition occurs is the percolation threshold [43,44].

The resistivity of the CNT/PANI/WEP composite coating loaded with 3.5% CNT/PANI composite filler is shown in Figure 11c. The results show that the conductivity of the composite coating is the best when the additional amount of AN is 15% of CNT during the preparation of the CNT/PANI composite. It is evident that PANI content increases in CNT/PANI/WEP composite coatings as the amount of AN increases, so the resistivity of composite coatings continues to decrease. The resistivity of CNT/PANI/WEP composite coating is as low as 2.2 × 10^4^ Ω·cm when AN mass is 15% of CNT. However, resistivity rebounds with a further increase in PANI content. This phenomenon may be caused by the following factors: Coated with PANI on the surface improves the dispersion uniformity of CNTs in the composite coating by enhancing interfacial adhesion between CNTs and the polymer matrix [45]. Simultaneous, coated with PANI on the surface of CNT, a one-dimensional material, also decreases the contact resistance between CNTs. Therefore, improvement of filler dispersion and reduction in contact resistance between the fillers result in lower resistivity of the composite coating. However, an excess of PANI leads to the agglomeration of CNTs, which negatively impacts the conductivity of the filler, just as demonstrated in Figure 5(d1,d2) and Figure 6(d1,d2).

The resistance of the GO/CNT/PANI/WEP composite coating loaded with 3.5% GO/CNT/PANI of different GO components is shown in Figure 11d. It is proven that adding an appropriate amount of GO to the filler has no adverse effect on the resistivity of the coating. According to the diagram, adding a paucity of GO to the composite coating does not significantly alter its resistivity. However, the resistivity of the coating is increased with an excess of GO added. Combined with the analysis of Figure 7 and Figure 8, it can be concluded that the dispersion of the filler in the water and coating and the corrosion resistance of the coating were improved by introducing GO into the composite coating. GO has excellent anticorrosive properties, but it tends to agglomerate and has high resistivity. Wrapping PANI on GO could compensate for its high resistivity shortcoming of GO. Therefore, the addition of GO improves the corrosion resistance of the composite coating without negatively affecting the electrical conductivity of the composite coating. However, PANI is not uniformly distributed on GO when GO has overdosed. The barrier effect of CNT on GO lamellae also becomes worse, which leads to a decrease in the dispersion and conductivity of the composite filler, as well as an increase in the resistivity of the composite coating.

### 3.6. Mechanical Performances Measurement

As a functional coating, the mechanical performances are also important besides the electrical conductivity and anticorrosive properties. The obtained mechanical performance data of different coatings are listed in Table 3, including flexibility, adhesion, pencil hardness, and impact resistance. The results show that the addition of CNT or CNT/PANI into WEP coatings could enhance the pencil hardness of coatings but have a negative effect on flexibility and impact resistance. As introducing an appropriate amount of GO during the preparation of GO/CNT/PANI composite, the GO/CNT/PANI composite filler exhibits more uniform distribution within the coating than CNT and CNT/PANI and results in better comprehensive properties. The adhesion of GO/CNT/PANI/WEP composite coating increased to grade 0, and the flexibility reached 5 mm while maintaining pencil hardness of 2H and impact resistance of 50 cm.

### 3.7. Antiorrosion Tests

#### 3.7.1. The Electrochemical Impedance Spectroscopy

**Figure 12:**
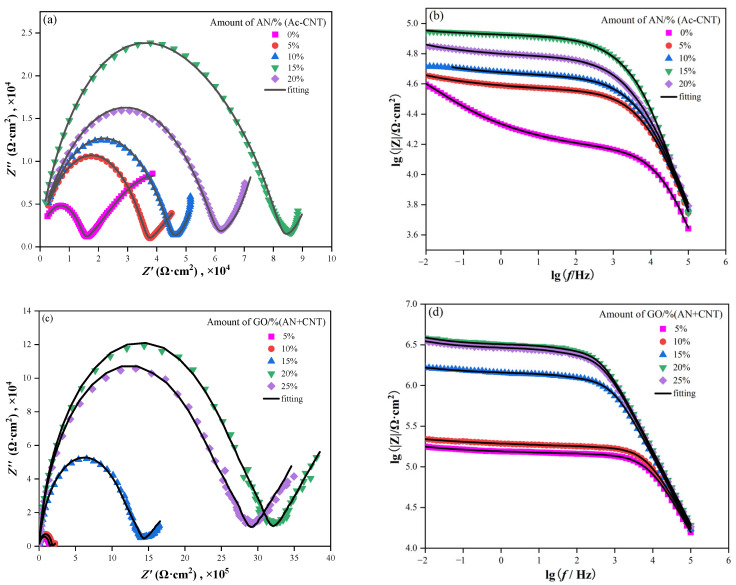
Nyquist and Bode plots of (**a**,**b**) CNT/PANI/WEP coating and (**c**,**d**) GO/CNT/PANI/WEP coating in 3.5 wt% NaCl solution.

In the Nyquist plots of the electrochemical impedance spectroscopy (EIS), a larger capacitive loop represented the better anticorrosion performance of the coatings [46]. It can be seen from Figure 12a that all the semicircle diameters of capacitive loops of CNT/PANI/WEP coatings are greater than that of CNT/WEP coating, and their semicircle diameters of capacitive loops increase as the content of PANI in the filler increases until the mass ratio of AN to CNT is 15%, of which the semicircle diameters of capacitive loops are the largest. The semicircle diameters of capacitive loops become smaller as continuing to increase the content of PANI. The decrease in resistance may be related to the agglomeration of PANI. Add GO while keeping the ratio of AN to the CNT unchanged, and the semicircle diameters of capacitive loops of GO/CNT/PANI/WEP composite coating become greater again than that of CNT/PANI/WEP coating and increase with the increase in the proportion of GO in the filler until the addition amount of GO is 20% of CNT and AN, of which the semicircle diameters of capacitive loops are the largest, as shown in Figure 12c, indicating that the barrier properties of the GO/CNT/PANI/WEP composite coating were better than that of the CNT/PANI/WEP composite coating, and the best when the addition amount of GO is 20% of CNT and AN.

In addition, the module of electrochemical impedance at 0.01 Hz (|Z|_0.01 Hz_) in the Bode plot in the EIS is a useful parameter for the characterization of the corrosion protection of composite coatings. Generally speaking, the higher |Z|_0.01 Hz_ means the better anticorrosion properties [47]. Compare Figure 12b,d, the |Z|_0.01 Hz_ of GO/CNT/PANI/WEP composite coating rapidly increased by one order of magnitude to 10^6^ after adding GO during the preparation of GO/CNT/PANI composite. The result suggests that the GO/CNT/PANI/WEP composite coating is more corrosion resistant than the GO/CNT/PANI composite coating for Q235 steel.

**Figure 13:**
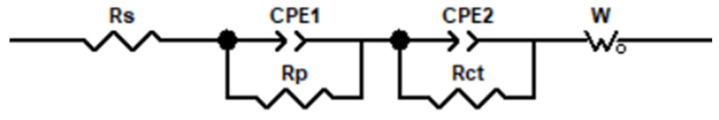
Equivalent circuit model for matching with the electrochemical impedance data.

To further analyze EIS results, the corresponding equivalent circuits (Figure 13) are established by Zview software (3.0.0.14, Scribner Associates Inc., Charlottesville, VA, USA) to fit the EIS data, and the obtained electrochemical parameters are listed in Table 4. R_s_ represents the resistance of the electrolyte solution, W stands for the Warburg diffusion element [48], R_p_ represents the pore resistance [49], R_ct_ represents the charge transfer resistance [50], and CPE1 and CPE2 are the constant phase elements [51]. R_ct_ reflects the protective performance of the coating on the metal; a higher R_ct_ value represents fewer corrosion reactions on the steel. R_p_ reflects its compactness, and a higher R_p_ value denotes a denser coating [52]. Based on the acquired data from EIS measurements (Table 4), The R_ct_ and R_p_ of the GO/CNT/PANI/WEP coating increase continuously with increasing GO content and maximize at 20% GO in the filler. Further increasing the GO content in the filler, the R_ct_ and R_p_ of the coating appeared to decrease. This may be caused by uneven dispersion of the filler in the coating. To be specific, the unique lamellar structure gives GO corrosion resistance while causing it to agglomerate easily [53]. Moreover, CNTs have a weakening effect on the blocking effect of GO when the content of GO is too much, and excessive GO causes agglomeration, which will harm the dispersion of the filler in the WEP coating [54]. The decrease in R_p_ also indicates a deterioration in the densification of the coating, which is the main reason for the decrease in the corrosion protection of the coating. The results show that the corrosion resistance of GO/CNT/PANI/WEP is the best compared with that of pure WEP, CNT/WEP, and CNT/PANI/WEP. At the same time, the composite coating has the best corrosion resistance when the content of GO is 20% of CNT and AN.

#### 3.7.2. Potentiodynamic Polarization Curves

**Figure 14:**
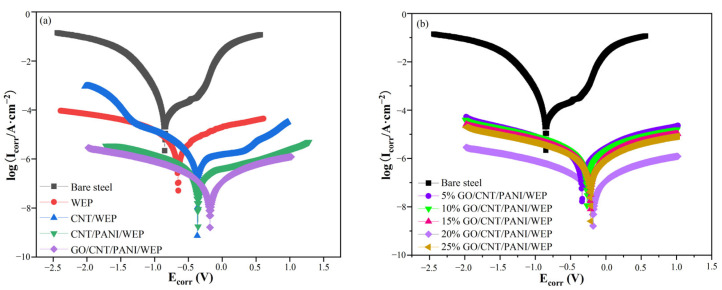
(**a,b**) Potentiodynamic polarization curves of the coated steel immersion in a 3.5 wt% NaCl solution.

Figure 14 shows the polarization curves of bare steel, coated steels with WEP coating, and composite coatings with different fillers immersed in a 3.5% NaCl solution. In general, corrosion potential (E_corr_) represents the sensitivity of a substrate surface to corrosion, while the anodic dissolution of metal ions and intensity of the cathodic oxygen reduction are typically expressed by corrosion current density (I_corr_). Thus, the smaller the I_corr_ and the more positive the E_corr_, the better the corrosion protection of the coating [55]. To obtain the electrochemical corrosion parameters, the polarization curves were fitted using standard extrapolations [56], and the value of polarization resistance (R_p_) was calculated by the Stern-Geary equation [57]. The results of the fitting of Figure 14a are shown in Table 5. Bare steel had an E_corr_ of −0.953 V and a WEP coating of −0.670 V. The E_corr_ of the coating increased significantly with the addition of CNT to WEP coating. Furthermore, the I_corr_ of the WEP coating drops by an order of magnitude with the addition of PANI to the filler. Among them, GO/CNT/PANI/WEP coatings have the best corrosion protection properties, with all other conditions remaining unchanged, excluding the addition of GO to filler. The following reasons mainly cause this phenomenon: To a certain extent, the CNTs could compensate for the defects produced in the curing process of WEP and improve the anticorrosive properties of the WEP coating [58]. Conductive PANI is a kind of excellent conductive anticorrosive filler, which has a certain degree of passivation of the metal and can be used in the formation of a layer of dense oxide film on the surface of the metal to prevent the corrosive substance invasion so that the corrosion rate of metal coated with composite coating containing PANI is significantly reduced [59]. GO is one of the most widely used and effective anticorrosion fillers. Its large specific surface area can increase the diffusion path of corrosive media in the coating, which can significantly improve the anticorrosion performance of the composite coating [60].

The fitting results of Figure 14b are shown in Table 6. It can be seen that the percentage of GO in the filler has a significant influence on the anticorrosion performance of the coating when the ternary composite material is used as filler. In the beginning, the anticorrosion performance of the composite coating will be improved with the increase in the amount of GO additions, and the corrosion current density of the coating is the lowest of 4.53 × 10^−8^ A·cm^−2^ with the GO content of CNTs and AN mass of 20%. The corrosion potential is the most positive, −0.180 V. At the same time, the corrosion rate is only 3.5 × 10^−4^ mm·a^−1^, which fell by order of magnitude compared with the neat WEP emulsion coatings. Continue to increase the ratio of GO in the filler; the composite coatings, on the contrary, appeared to be a decline in the performance of the coating. These results are closely related to the dispersion uniformity of the filler in the WEP coatings. The unique two-dimensional structure of GO gives it good anticorrosive properties; however, the strong van der Waals forces between GOs are prone to agglomeration, which negatively affects the various properties of the coating [61]. There is an excellent synergistic effect between them when CNTs and GO are used together, and CNTs can be interspersed between GO layers to block the agglomeration of GO. However, this blocking effect will decrease if the GO content is too high. Agglomeration of filler can lead to uneven distribution of fillers in WEP coatings and reduce the performance of the coatings [62]. The results show that the corrosion resistance of GO/CNT/PANI/WEP is the best compared with that of neat WEP, CNT/WEP, and CNT/PANI/WEP, and the composite coating has the best corrosion resistance when the content of GO in the filler is 20%.

#### 3.7.3. Salt Spray Test

**Figure 15:**
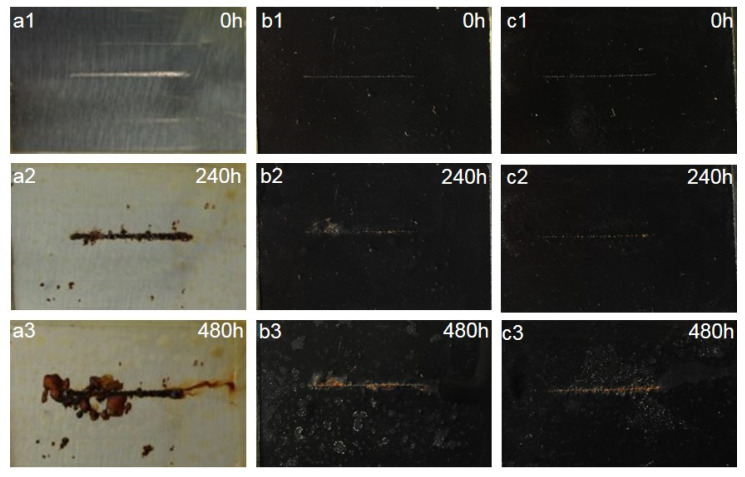
Optical photographs of (**a1**–**a3**) neat WEP coating and composite coating of (**b1**–**b3**) 3.5 wt% CNT/PANI/WEP and (**c1**–**c3**) 3.5 wt% GO/CNT/PANI/WEP before and after 240 h and 480 h of salt spray test.

A salt spray test was used to investigate the long-term corrosion protection properties of WEP, CNT/PANI/WEP, and GO/CNT/PANI/WEP composite coatings. Figure 15 shows the optical images of the samples before and after being exposed to the salt spray apparatus at different times, from which it can be seen that the neat WEP coating showed a more obvious corrosion phenomenon when exposed to the salt spray for 240 h. At the same time, the coating was whitened due to the absorption of moisture in the coating during the erosion of the salt spray experiments. The neat WEP coating showed severe corrosion after the coating was placed in the salt spray apparatus for 480 h. At the same time, a severe corrosion diffusion phenomenon was observed around the scratches. Compared to the neat WEP coating, the CNT/PANI/WEP composite coating showed slight corrosion of the metal substrate after 240 h in the salt spray apparatus and the time in the salt spray apparatus up to 480 h. White spots appeared on the surface of the composite coating, and the area around the scratches showed the spread of minor rust. The comparison of CNT/PANI/WEP composite coating and GO/CNT/PANI/WEP composite coating shows that there is almost no corrosion in the scratched area of GO/CNT/PANI/WEP composite coating when it is exposed to salt spray for 240 h, and a small amount of rust appeared after it was exposed to salt spray for 480 h, and there is no obvious corrosion-spreading phenomenon. The results showed that the addition of the CNT/PANI nanofiller to the neat WEP coating improved the corrosion resistance of the WEP coating, and the addition of GO/CNT/PANI nanofiller, which introduced GO to the CNT/PANI nanofiller during preparation, to the neat WEP coating significantly improved the anticorrosion performance of the coating again. This phenomenon is mainly attributed to the fact that the nanofillers compensate for the microporous defects generated during the curing process of the WEP and the barrier effect of GO on the diffusion of corrosive media [63,64].

### 3.8. Corrosion Protection Mechanism

**Figure 16:**
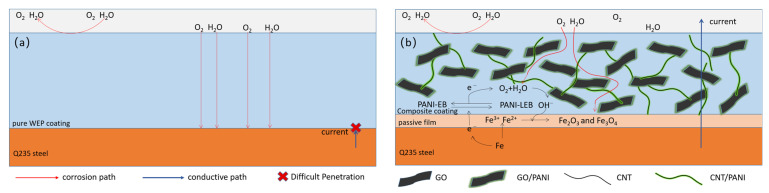
Schematic illustration of (**a**) neat WEP coating and (**b**) GO/CNT/PANI/WEP composite coating during the corrosion process.

Schematic illustrations of the coating systems are depicted in Figure 14 to further discuss the conductivity and anticorrosion mechanism of the WEP and GO/CNT/PANP/WEP coatings. The barrier performance of neat WEP coating for corrosive media is limited, and corrosive media can easily reach the surface of the metal, resulting in corrosion of the metal. Moreover, neat WEP does not have conductive properties, so the current cannot be evacuated through the coating to the outside of the system, as shown in Figure 16a.

The anticorrosion and conductive properties of the composite coatings were mainly attributed to the following effects, based on the analysis of the characterization of the GO/CNT/PANI composite fillers in conjunction with the test results of the anticorrosion properties of the composite coatings. Firstly, the synergistic effect of GO, PANI, and CNTs makes the ternary composite material uniformly dispersed in the WEP coating, and the lamellar structure of the composite material can impede the invasion of corrosive media and prolong the path of corrosive media to reach the metal surface, thus protecting the metal. Moreover, the PANI can capture the electrons from the dissolved metal and transform it into a leucoemeraldine base (LEB) from an emeraldine base (EB) due to its electrochemical activity. The ferric ions are directly oxidized by oxygen to form the ferric oxide and ferric tetroxide, and then the LEB can be oxidized to EB to continue the oxidation process, and a dense oxidative film can be formed on the surface of the metal to protect it through this benign cycle [65]. Furthermore, PANI coating on the oxidized GO surface can compensate for the poor conductivity of GO. CNTs coated with PANI interspersed between GO sheets to form a three-dimensional conductive network while preventing agglomeration between graphene sheets [66]. Direct current can flow from the metal to the outside of the system through this three-dimensional conductive network, as shown in Figure 16b.

In conclusion, the corrosion resistance and electrical conductivity of the composite coating have been improved due to the synergistic effect of GO, PANI, and CNTs, including uniformly dispersed stability, dense metal oxide layer, and three-dimensional network structure.

## 4. Conclusions

To improve the electrical conductivity and anticorrosion properties of waterborne epoxy coating simultaneously, GO/CNT/PANI nanocomposites were successfully synthesized as an ideal filler. The characterization by FT-IR, SEM, TEM, and XRD proved that PANI is attached to the surface of CNT and GO and forms a cross-linked three-dimensional structure. The conductive anticorrosive properties and mechanism of the WEP composite coatings filled with GO/CNT/PANI nanocomposites were systematically studied by resistivity measurement, EIS, polarization curves, and salt spray experiments. The results showed that the GO/CNT/PANI/WEP coating had the best corrosion resistance, with the positive corrosion potential (−0.180 V) shifted compared with that of the neat WEP, CNT/WEP, and CNT/PANI/WEP coating. The GO/CNT/PANI/WEP coating has a corrosion current density of 4.53 × 10^−8^ A·cm^−2^ and a corrosion rate of 3.50 × 10^−4^ mm·a^−1^, which is an order of magnitude lower compared to the CNT/WEP coating and the neat WEP coating. The protection efficiency also increased to 99.72%. In addition, 3.5 wt% GO/CNT/PANI/WEP coating showed only slight corrosion of composite coatings after the salt spray test for 480 h. The synergistic effect of GO, CNT, and PANI leads to improved dispersion of nanofiller in the coating and the formation of a three-dimensional conductive network within the coating. This network reduces the electrical resistivity of the composite coating to only 2.3 × 10^4^ Ω·cm, which is much lower than the required resistivity of 10^6^ Ω·cm. Our findings developed an anticorrosive coating with conductive properties based on a new type of GO/CNT/PANI nanocomposites and broadened the application scope of polymer coatings, particularly where the protection of lightning rods, oil storage tanks, grounding grids, and so on.

## Figures and Tables

**Table 1:** Formulations of CNT/WEP composite coatings.

Samples	A1	A2	A3	A4	A5	A6	A7	A8	A9	A10	A11
CNT/g	0.02	0.04	0.06	0.08	0.10	0.12	0.14	0.16	0.18	0.20	0.24
WEP/g	4.0	4.0	4.0	4.0	4.0	4.0	4.0	4.0	4.0	4.0	4.0
CNT/WEP/%	0.5	1.0	1.5	2.0	2.5	3.0	3.5	4.0	4.5	5.0	6.0

**Table 2:** Formulations of CNT/PANI/WEP and GO/CNT/PANI/WEP composite coatings.

Samples	B1	B2	B3	B4	C1	C2	C3	C4	C5
CNT/g	0.133	0.127	0.122	0.117	0.116	0.111	0.106	0.101	0.097
AN/g	0.007	0.013	0.018	0.023	0.017	0.017	0.016	0.015	0.015
GO/g	-	-	-	-	0.007	0.013	0.018	0.023	0.028
WEP/g	4.0	4.0	4.0	4.0	4.0	4.0	4.0	4.0	4.0
AN/CNT/%	5.0	10.0	15.0	20.0	15.0	15.0	15.0	15.0	15.0
GO/(AN + CNT)/%	-	-	-	-	5.0	10.0	15.0	20.0	25.0
Fillers/WEP/%	3.5	3.5	3.5	3.5	3.5	3.5	3.5	3.5	3.5

**Table 3:** Mechanical performances of coatings.

Samples	WEP	CNT/WEP	CNT/PANI/WEP	GO/CNT/PANI/WEP
Flexibility/mm	3	10	10	5
Pencil hardness (level)	2H	3H	3H	2H
Adhesion (level)	1	1	1	0
Impact resistance/cm	50	40	40	50

**Table 4:** Equivalent circuit parameters fitting with the acquired data from EIS measurements.

Sample	GO/%	R_s_/Ω·cm^2^	R_ct_/Ω·cm^2^	CPE_1_		R_p_/Ω·cm^2^	CPE_2_	
				Y_0_/Ω·cm^−2^·s^n^	*n*		Y_0_/Ω·cm^−2^·s^n^	*n*
C1	5	2.82 × 10^2^	9.37 × 10^4^	6.32 × 10^−10^	0.89	4.80 × 10^4^	6.67 × 10^−9^	0.76
C2	10	3.24 × 10^2^	1.72 × 10^5^	5.36 × 10^−10^	0.87	1.40 × 10^5^	2.72 × 10^−5^	0.29
C3	15	2.78 × 10^1^	1.15 × 10^6^	5.16 × 10^−10^	0.73	2.31 × 10^5^	5.16 × 10^−8^	0.90
C4	20	2.40 × 10^1^	2.61 × 10^6^	2.63 × 10^−10^	0.92	5.13 × 10^5^	3.27 × 10^−8^	0.78
C5	25	2.46 × 10^1^	2.31 × 10^6^	2.95 × 10^−10^	0.91	4.12 × 10^5^	3.07 × 10^−8^	0.77

**Table 5:** Tafel fitting data of bare steel, WEP, CNT/WEP, CNT/PANI/WEP, and GO/CNT/PANI/WEP.

Sample	b_a_	b_c_	E_corr_/V	I_corr_/A·cm^−2^	CR/mm·a^−1^	R_p_/Ω·cm^2^	PE/%
Bare steel	1.83	0.12	−0.953	1.24 × 10^−4^	9.59 × 10^−1^	3.99 × 10^2^	-
WEP	0.84	0.44	−0.670	3.73 × 10^−6^	2.88 × 10^−2^	3.38 × 10^4^	77.39
CNT/WEP	0.41	0.28	−0.363	6.32 × 10^−7^	4.89 × 10^−3^	1.14 × 10^5^	96.17
CNT/PANI/WEP	0.32	0.28	−0.356	9.67 × 10^−8^	7.40 × 10^−4^	6.99 × 10^5^	99.41
GO/CNT/PANI/WEP	0.34	0.35	−0.180	4.53 × 10^−8^	3.50 × 10^−4^	1.65 × 10^6^	99.72

**Table 6:** Tafel fitting data for GO/CNT/PANI/WEP composite coatings with different GO contents.

Sample	GO/%	b_a_	b_c_	E_corr_/V	I_corr_/A·cm^−2^	CR/mm·a^−1^	R_p_/Ω·cm^2^	PE/%
Bare steel	-	1.83	0.12	−0.953	1.24 × 10^−4^	9.59 × 10^−1^	3.99 × 10^2^	-
C1	5	0.55	0.47	−0.344	8.50 × 10^−7^	6.57 × 10^−3^	1.29 × 10^5^	94.85
C2	10	0.52	0.46	−0.265	7.93 × 10^−7^	6.13 × 10^−3^	1.34 × 10^5^	95.19
C3	15	0.45	0.43	−0.218	4.81 × 10^−7^	3.72 × 10^−3^	1.99 × 10^5^	97.08
C4	20	0.34	0.35	−0.180	4.53 × 10^−8^	3.50 × 10^−4^	1.65 × 10^6^	99.72
C5	25	0.34	0.36	−0.214	2.81 × 10^−7^	2.17 × 10^−3^	2.72 × 10^5^	98.29

## Data Availability

All data generated or analyzed during this study are included in this published article and its Supplementary Information Files.

## Supplementary Materials

The following supporting information can be downloaded at: https://www.mdpi.com/article/10.3390/polym16182641/s1, Figure S1: FT-IR spectra of PANI, Ac-CNT, and CNT/PANI; Figure S2: XRD patterns of GO, PANI, and Ac-CNT.
