# Supplementary figures and images for: Preparation and Characterization of Graphene Oxide/Carbon Nanotube/Polyaniline Composite and Conductive and Anticorrosive Properties of Its Waterborne Epoxy Composite Coatings
Source: Polymers (Basel). 2024 Sep 19;16(18):2641. doi: 10.3390/polym16182641 (PMC11435755; doi:10.3390/polym16182641)

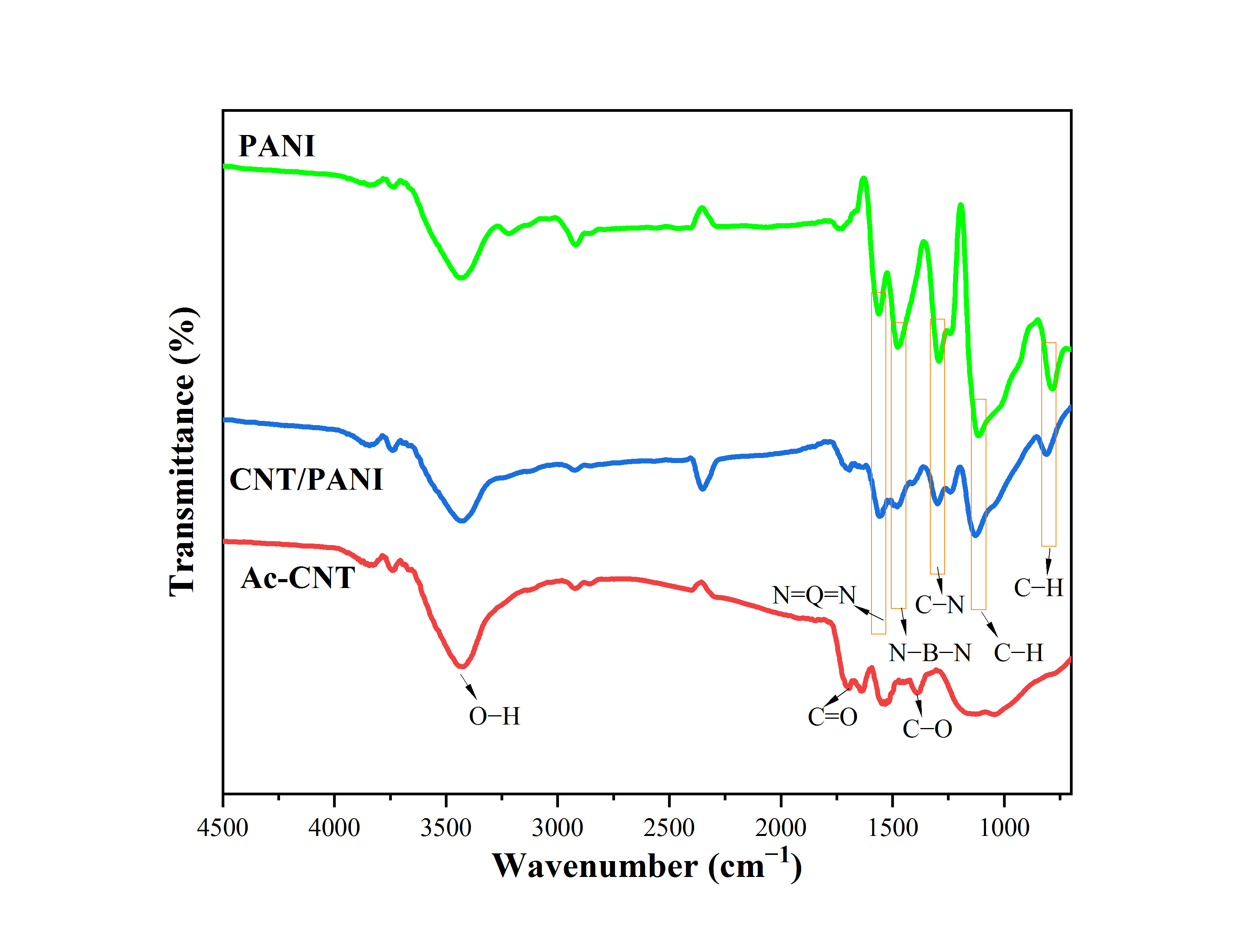

Supplement: Supplementary file 1 [file polymers-16-02641-s001.zip › Figure S1 FT-IR spectra of PANI CNT CNTPANI.tif]

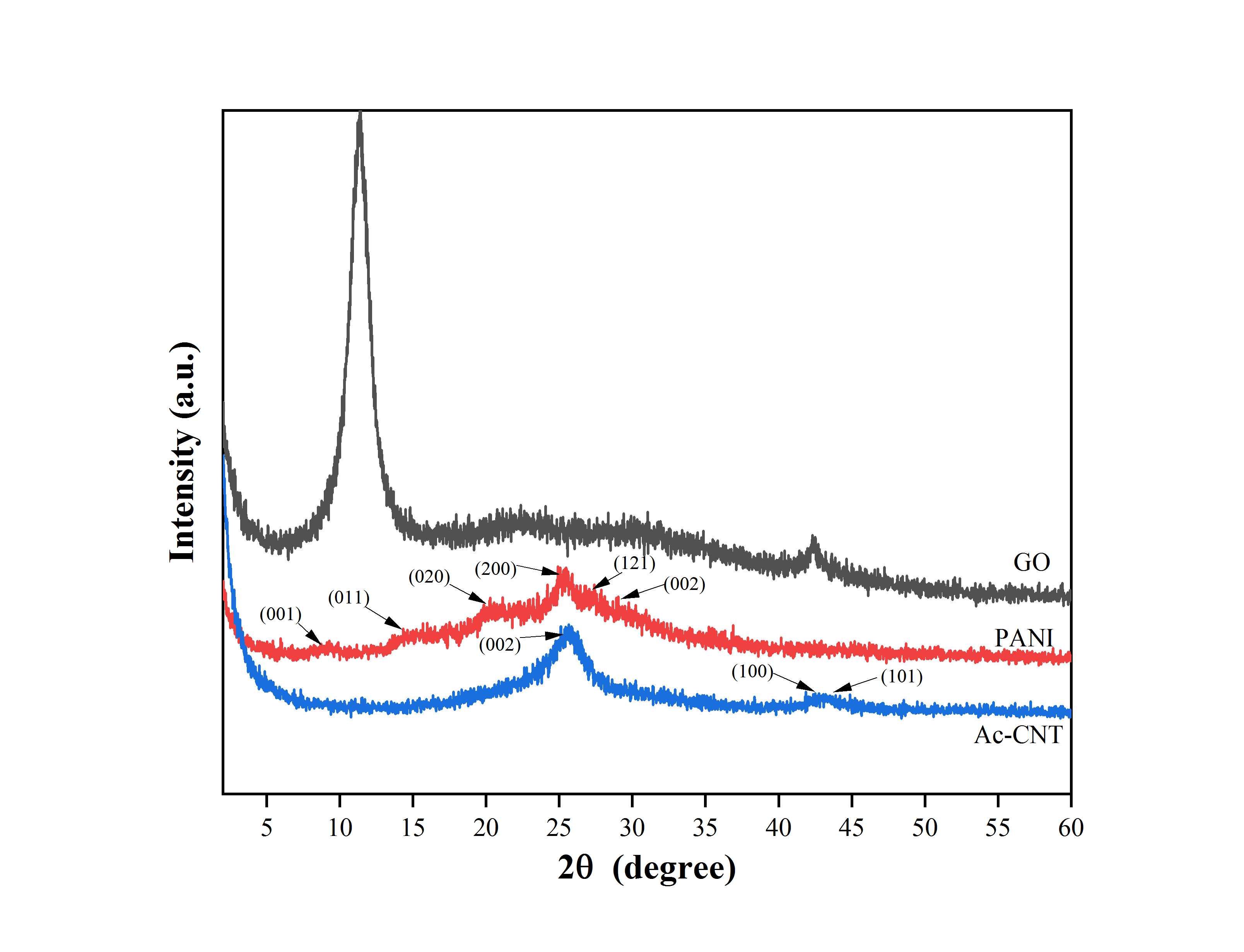

Supplement: Supplementary file 1 [file polymers-16-02641-s001.zip › Figure S2 XRD patterns of CNT PANI GO.tif]
